# Direct bare metal needle puncture and balloon angioplasty in calcified plaques of the common femoral artery guided by angiography (“BAMBOO SPEAR”)

**DOI:** 10.1186/s42155-021-00217-7

**Published:** 2021-03-04

**Authors:** Naoki Hayakawa, Satoshi Kodera, Masataka Arakawa, Satoshi Hirano, Sandeep Shakya, Junji Kanda

**Affiliations:** 1 Department of Cardiovascular Medicine, Asahi General Hospital, I-1326 Asahi, Chiba, 289-2511 Japan; 2 Department of Cardiovascular Medicine, University of Tokyo Hospital, Tokyo, Japan

**Keywords:** Endovascular therapy, Needle, Common femoral artery, Calcified plaque

## Abstract

**Background:**

Surgical endarterectomy for common femoral artery (CFA) disease is still considered the gold standard for treatment. Development of various techniques and devices has improved the clinical results of endovascular therapy (EVT) for CFA. However, severe conditions remain, especially for occlusive lesions owing to calcified plaque. We developed a useful technique for passing a lesion by directly penetrating the calcified plaque of the CFA using a bare metal needle and then passing through a balloon or dilating it. We named this technique “direct bare metal needle puncture and balloon angioplasty in calcified plaques of the common femoral artery guided by angiography” or “BAMBOO SPEAR.”

**Main text:**

This report describes our technique for crossing a lesion by directly penetrating the calcified plaque of the CFA using a needle. We report a case of a 73-year-old male with hemodialysis who presented with cyanosis and ischemic rest pain of both lower limbs. Control angiography showed total occlusion of the left CFA with a calcified plaque. We advanced a 21-G metal needle that was slightly curved into the blood vessel from where the lumen of the distal CFA was located. The needle was advanced into the center of the calcified plaque, while observing from multiple directions with a fluoroscopic guide. We succeeded in advancing the needle into the lumen of the distal external iliac artery. After guidewire crossing, intravascular ultrasound (IVUS) showed that guidewire was able to completely pass through the center of the calcified plaque. We could dilate the lesion by scoring balloon and drug-coated balloon. The final angiography showed sufficient results. We named this technique “direct BAre Metal needle puncture and BallOOn angioplaSty in calcified PlaquEs of the common femoral ARtery guided by angiography” (BAMBOO SPEAR).

**Conclusions:**

The BAMBOO SPEAR technique may be considered a useful option in EVT for occlusive CFA with calcified plaques.

## Background

Development of endovascular therapy (EVT) led to one of the first-line treatment strategies for peripheral artery disease (PAD) (Norgen et al. 2007). However, surgical endarterectomy is still considered as the gold standard treatment in common femoral artery (CFA) disease because of favorable long-term durability (Nishibe et al. 2015). However, postoperative morbidity, including wound infection and lymphatic leakage, can occur after this surgical approach (Nguyen et al. 2015). Recently, feasible clinical results of stenting for the CFA or EVT for the CFA using a drug coated balloon (DCB) have been reported (Kuo et al. 2019; Gouëffic et al. 2017). However, if severe calcified lesions or eccentric calcified plaques are present, dilatation is often difficult. Excessive dilation may lead to a risk of vascular perforation, which leads to the need for using stent-grafts. We sometimes intentionally advance the extra hard tip of a guidewire through the center of calcification, but it can be difficult for severe calcifications. The use of atherectomy devices can be useful, but there are also problems with this device, such as the fact that some areas do not cover medical insurance for this device, the high cost, and complexity of the procedure (Stavroulakis et al. 2018). We developed a useful and simple technique for crossing a lesion by directly penetrating the calcified plaque of the CFA using a bare metal needle, which then passes through the balloons or dilates it. After passing through the guidewire, externalization of the guidewire is performed, and then a bougie is used with a needle so that balloons can pass through relatively easily. Because the guidewire can pass through the center of a calcified plaque, the risk of vascular injury is small and the balloon can be well dilated, and an acceptable initial lumen gain is easy to obtain. We named this technique “direct BAre Metal needle puncture and BallOOn angioplaSty in calcified PlaquEs of the common femoral ARtery guided by angiography” (BAMBOO SPEAR). And we present here a case of using this BAMBOO SPEAR technique for total occlusion of the CFA with calcified plaques.

## Main text

**Fig. 1:**
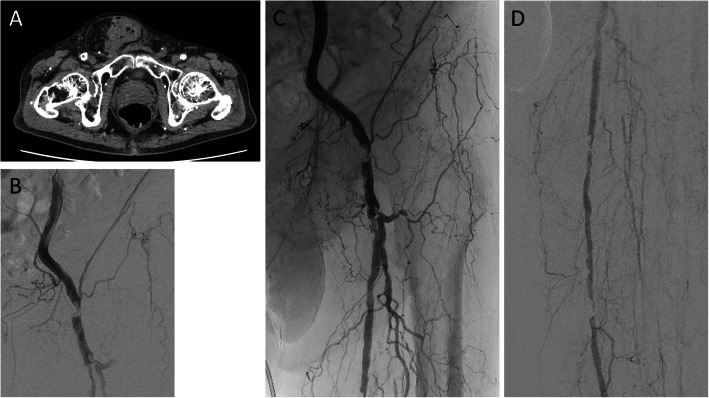
**a** Plain computed tomography scan shows a calcified plaque in the left CFA. **b–d** Control angiography shows total occlusion of the left CFA with a calcified plaque and total occlusion of the middle to distal SFA

**Fig. 2:**
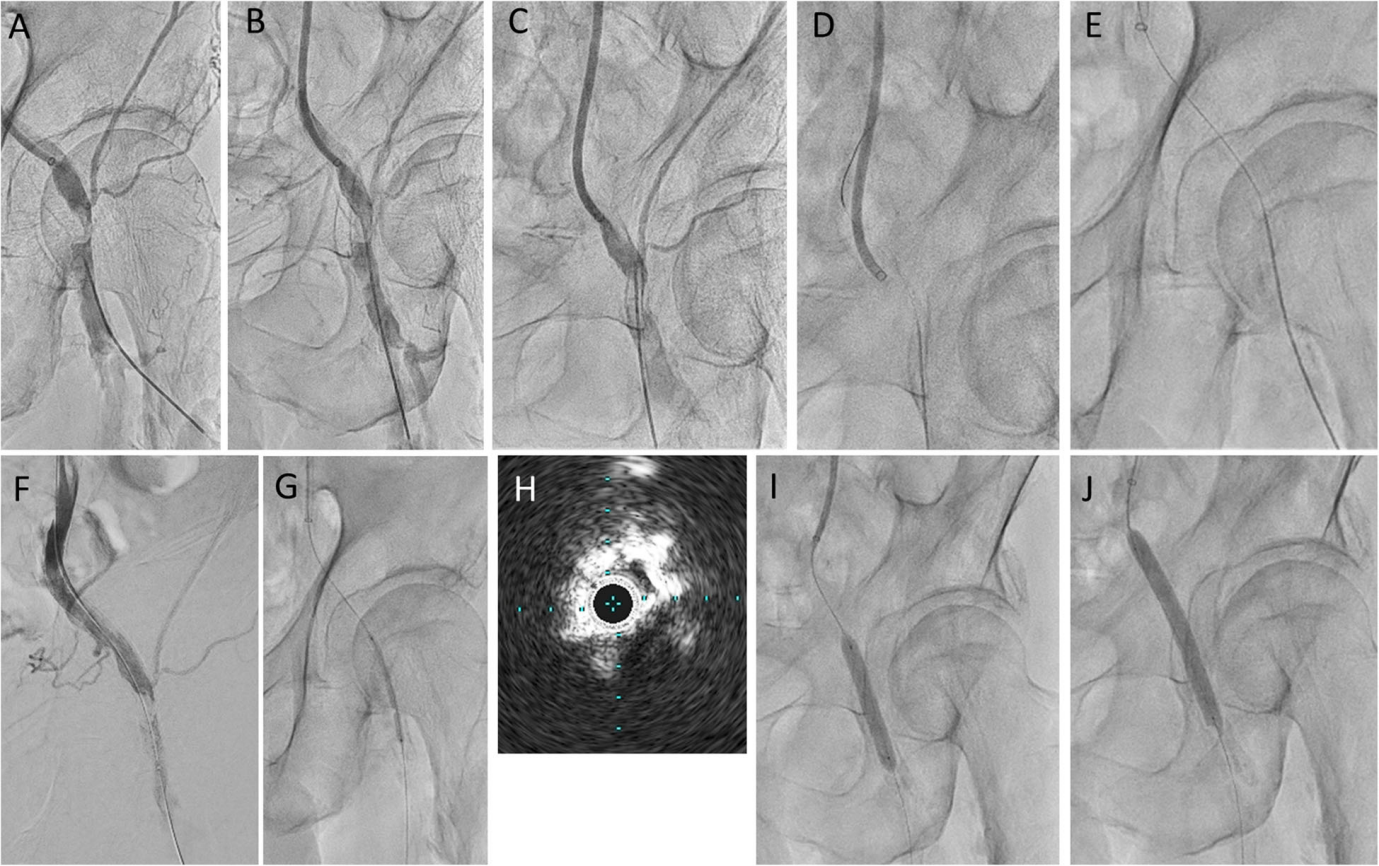
**a, b** Direct puncture from the distal CFA. **c** We succeeded in advancing the needle as it was and into the lumen of the distal external iliac artery. **d–f** A 0.014-in. guidewire was inserted from behind the needle and the guidewire was advanced into the guiding sheath to make it externalized. **g** We dilated a 3.0 × 40-mm balloon. **h** IVUS shows the guidewire passed through the center of a calcified plaque. **i, j** We dilated the scoring balloon and drug-coated balloon

**Fig. 3:**
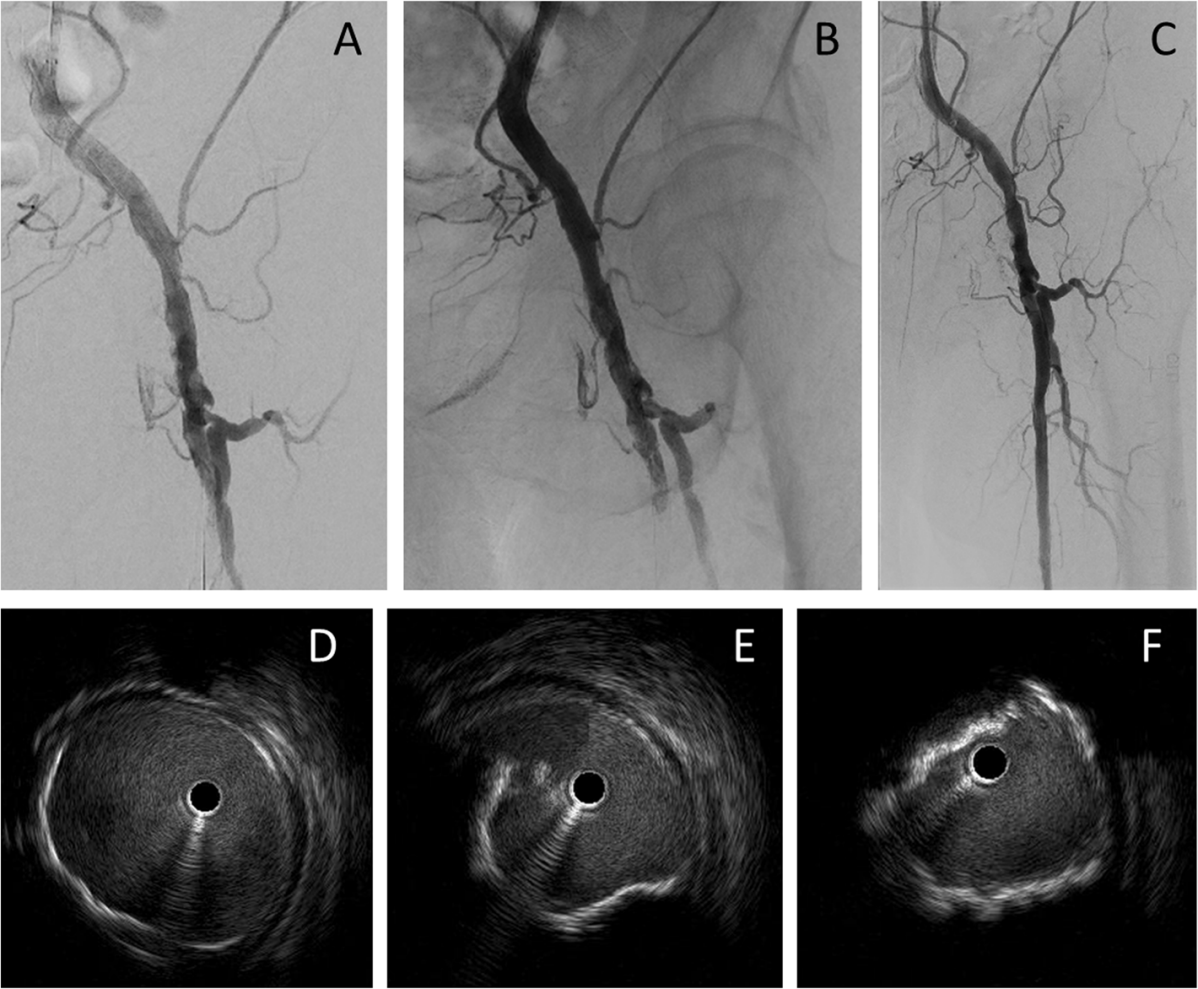
**a, b** Final angiography shows sufficient expansion of the lesions. **c** Angiography when the SFA was treated 5 months after CFA treatment. The CFA was well opened. **d–f** IVUS shows that the CFA is sufficiently patent. Panel **d** shows the proximal CFA, panel **e** shows the mid-CFA, and panel **c** shows the distal CFA

A 73-year-old man had hemodialysis owing to diabetes mellitus with coronary artery disease and old cerebral infarction. Cyanosis and ischemic rest pain of both lower limbs were observed. Preprocedural plain computed tomography showed severe calcification in the left femoral artery (Fig. 1a). A 6-Fr Destination guiding sheath was inserted into the right CFA via the contralateral approach. Control angiography showed total occlusion of the left CFA with a calcified plaque and total occlusion of the middle to distal SFA (Fig. 1b–d). Because calcification was remarkable, we decided to use a metal needle from the retrograde direction in primary. A 21-G metal needle (Merit Advance® angiography needle; Merit Medical) that was slightly curved was inserted into the blood vessel from where the lumen of the distal CFA was located. The needle was advanced into the center of the calcified plaque, while observing from multiple directions with a fluoroscopic guide (Fig. 2a, b). We succeeded in advancing the needle as it was and into the lumen of the distal external iliac artery (Fig. 2c). A 0.014-in. guidewire (Gladius MGES® guidewire; Asahi Intec) was inserted from behind the needle and the guidewire was advanced into the guiding sheath to enable its externalization (Fig. 2d–f). The calcified plaque was bougied several times with a metal needle that was coaxially placed on the externalization wire to perform modification of the lesion. After the antegrade re-wiring and pre dilatation, IVUS showed that the wire route was able to completely pass through the center of the calcified plaque (Fig. 2g, h). After dilation with a scoring balloon 6.0 × 40-mm (Lacrosse NSE® scoring balloon; Nippro) with intravascular hemostasis of the retrograde puncture site (Fig. 2i). Finally, we dilated a 6.0 × 60-mm drug-coated balloon (Lutonix RX® drug-coated balloon; BARD, AZ, USA) at the CFA lesion to prevent restenosis (Fig. 2j). The final angiography showed good results and the pressure gradient had disappeared completely (Fig. 3a, b). When the residual lesion of the SFA was treated 5 months after intervention of the CFA, the CFA was well opened, and IVUS also showed a sufficient lumen area (Fig. 3c–f).

### BAMBOO SPEAR technique

**Fig. 4:**
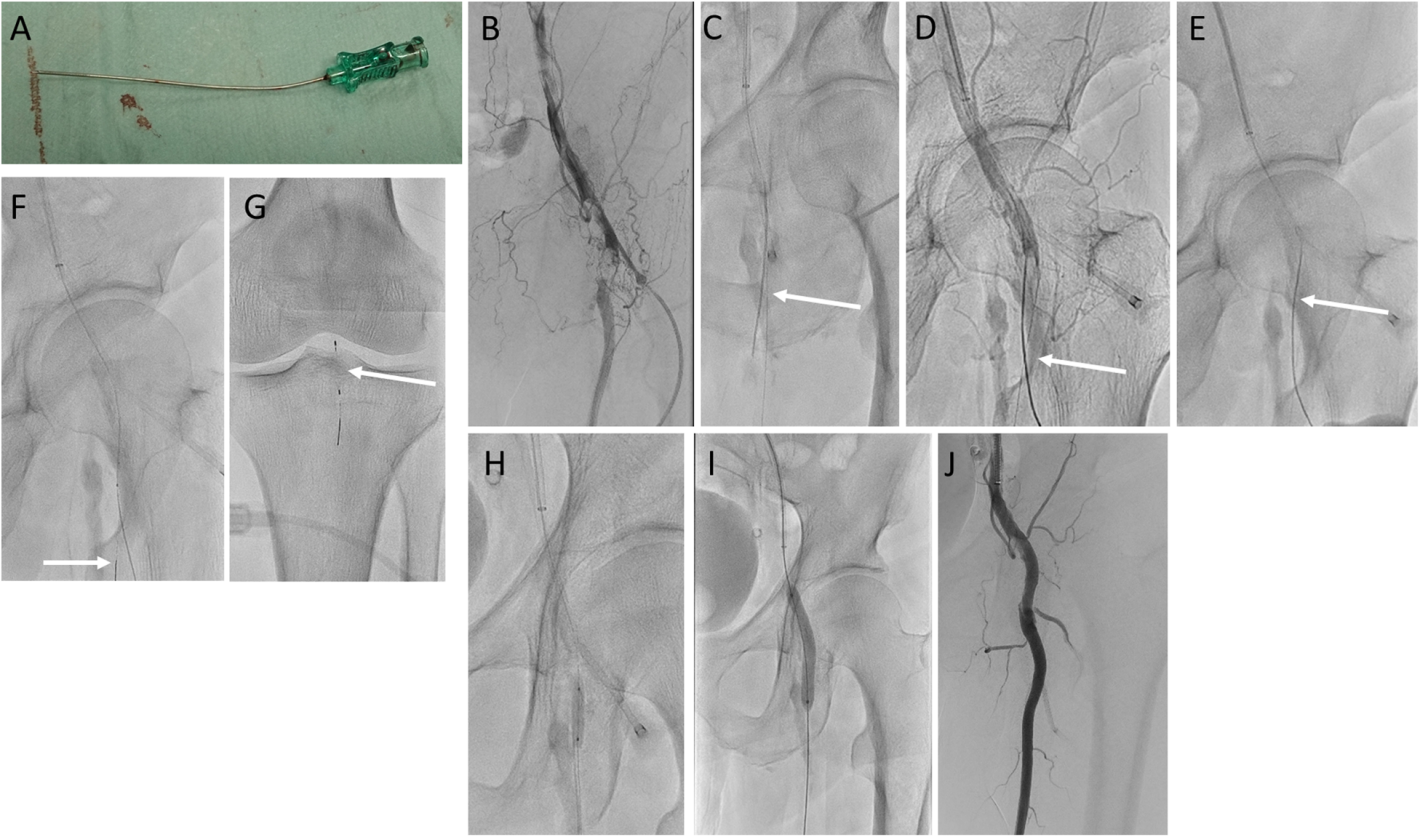
**a** Image showing a 21-G needle that is slightly curved. **b** Calcified plaque in the left CFA. **c, d** The needle was advanced into the center of the calcified plaque while observing from multiple directions with a fluoroscopic guide. **e** The needle on the externalized guidewire is coaxially placed in and out several times to puncture the lesion. **f** An antegrade wire was advanced into the SFA. **g** We placed the filter wire in the popliteal artery. **h, i** We dilated the CFA with a scoring balloon and high-pressure balloons. **j** Final angiography shows that the CFA is well opened

A slightly curved 21-G or 20-G metal needle was inserted from distal true lumen and then advanced into the center of the calcified plaque, while observing from multiple directions with a fluoroscopic guide (Fig. 4a–d). The metal needle on the externalized guidewire was then coaxially placed in and out several times to puncture the lesion (Fig. 4e, f). We then advanced another guidewire in the direction of the SFA. We usually placed a filter wire in the popliteal artery (Fig. 4g). The lesion was dilated with an antegrade balloon. At this time, the metal needle was removed, but by covering and expanding the puncture site, hemostasis could be achieved from inside the blood vessel at the same time (Fig. 4h). Using the results of angiography after balloon dilatation, we decided to finish with conventional balloon angioplasty alone, a DCB, or deploying a stent if recoil or dissection was severe (Fig. 4i, j).

## Discussion

We demonstrated the feasibility of EVT for CFA with severe calcified plaques using direct puncture of a metal needle. In this case, we were able to pass through the center of the calcified plaque and obtain sufficient initial lumen gain after balloon angioplasty by a simple method. Obtaining the initial lumen area is difficult if severe calcified lesions are present. Especially in the case of eccentric calcified nodules, not only does an open lumen not spread sufficiently, but excessive balloon dilation also involves the risk of vascular perforation. There has been a report on the feasibility of atherectomy devices (Stavroulakis et al. 2018). However, there is also the problem that these devices cannot be used in some areas for the reason that medical insurance does not cover them.

Passing the guidewire through heavy calcification is often difficult. Our method appears to be a simple and easy method of piercing a lesion with a metal needle and modifying the lesion. In the EVT field, methods using a metal needle, such as the PIERCE technique, and inner PIERCE technique for patients in whom there is difficulty in passing a balloon, have been reported (Ichihashi et al. 2014; Nakama et al. 2020). Metal needles are much harder and sharper than guidewires, and therefore, they can penetrate even with severe calcification. Our method is primarily to use a metal needle instead of guidewires to allow severe calcification to pass directly. Our method can also help subsequent balloon passage by modifying the lesion from intravessels, as well as passage of calcification.

We usually use a scoring balloon or cutting balloon after guidewire passage combined with high-pressure balloons.

Surgical endarterectomy appears to be a gold standard treatment for calcified CFA lesions. However, we consider that our method can be indicated for cases in which EVT should be chosen because of problems, such as the general condition and comorbidities. Additionally, our method can be indicated in cases in which performing EVT appears to be preferable for aortoiliac lesions and femoropopliteal lesions at the same time as CFA disease.

As a possible complication, the possibility of distal embolism cannot be ruled out because calcification expands while being crushed. Therefore, we usually try to place a filter wire (Parachute® filter wire; Keisei Medical, Tokyo, Japan) in the popliteal artery after passing the anterograde guidewire. Fortunately, we have not experienced any clinically problematic distal embolism. We always insert a guidewire into the antegrade guiding sheath to make the wire externalized. This procedure reduces the risk that the guidewire will be under tension and the needle will get caught. Enlarging the fluoroscopic image and attempting to proceed slowly at the beginning as much as possible are also important to avoid complications. Advancing carefully with the metal needle and guide wire as coaxially as possible is also recommended.

In our case, the initial success of the procedure and good short-term prognosis were confirmed, but the long-term patency is still unclear. A much larger study is required to confirm the safety and efficacy of our method. This procedure appears to be difficult to perform if the calcified plaques are continuous to the SFA and not just in the CFA, or if the SFA is completely occluded from just proximally. In such cases, a metal needle may be inserted directly from the distal part of the occluded CFA, only the CFA may pass through the calcified plaque by metal needle, and the remaining lesions may be passed through according to standard procedures. Even when the CFA to the external iliac artery (EIA) is continuously occluded, applying this technique appears to be difficult because the EIA has considerable bending and it is difficult to pass through it only with a needle. Additionally, applying this method to the ipsilateral up to the proximal SFA is possible. However, adapting to the direction of the mid-SFA or popliteal artery owing to the problem of needle length and angle is difficult. Although this technique may be applied to more peripheral arteries such as the dorsalis pedis artery, it seems that it is more difficult to pass the lesion by needle alone than CFA because of the small vessel diameter. To the best of our knowledge, this technique has not been previously reported. Therefore, more cases need to be accumulated and the efficacy and safety of this procedure need to be verified.

## Conclusions

We have developed a useful technique for directly penetrating calcified plaques of the CFA using a bare metal needle for passage of a balloon and dilation of lesions. We call this approach the BAMBOO SPEAR technique. The BAMBOO SPEAR technique could be a useful option in EVT for an occlusive CFA with a calcified plaque.

## Data Availability

The datasets used and/or analysed during the current study are available from the corresponding author on reasonable request.

## Abbreviations

CFA: Common femoral artery
EVT: Endovascular therapy
SFA: Superficial femoral artery
IVUS: Intravascular ultrasound
PAD: Peripheral artery disease
DCB: Drug coated balloon
CTO: Chronic total occlusion
EIA: External iliac artery
